# Large-roll growth of 25-inch hexagonal BN monolayer film for self-release buffer layer of free-standing GaN wafer

**DOI:** 10.1038/srep34766

**Published:** 2016-10-19

**Authors:** Chenping Wu, Abdul Majid Soomro, Feipeng Sun, Huachun Wang, Youyang Huang, Jiejun Wu, Chuan Liu, Xiaodong Yang, Na Gao, Xiaohong Chen, Junyong Kang, Duanjun Cai

**Affiliations:** 1 Fujian Key Laboratory of Semiconductor Materials and Applications, CI center for OSED, College of Physical Science and Technology, Xiamen University, Xiamen 361005, China; 2 Research Center for Wide-gap Semiconductors, State Key Laboratory for Artificial Microstructures and Mesoscopic Physics, School of Physics, Peking University, Beijing 100871, China; 3 State Key Laboratory of Physical Chemistry of Solid Surfaces, College of Chemistry and Chemical Engineering, Xiamen University, Xiamen 361005, China; 4 Department of Chemistry, Duke University, Durham, NC 27708-0354, USA

## Abstract

Hexagonal boron nitride (h-BN) is known as promising 2D material with a wide band-gap (~6 eV). However, the growth size of h-BN film is strongly limited by the size of reaction chamber. Here, we demonstrate the large-roll synthesis of monolayer and controllable sub-monolayer h-BN film on wound Cu foil by low pressure chemical vapor deposition (LPCVD) method. By winding the Cu foil substrate into mainspring shape supported by a multi-prong quartz fork, the reactor size limit could be overcome by extending the substrate area to a continuous 2D curl of plane inward. An extremely large-size monolayer h-BN film has been achieved over 25 inches in a 1.2” tube. The optical band gap of h-BN monolayer was determined to be 6.0 eV. The h-BN film was uniformly transferred onto 2” GaN or 4” Si wafer surfaces as a release buffer layer. By HVPE method, overgrowth of thick GaN wafer over 200 μm has been achieved free of residual strain, which could provide high quality homo-epitaxial substrate.

Hexagonal boron nitride (h-BN) has attracted broad attentions due to its similar honeycomb layer structure as graphene and moreover the wide band-gap (~6 eV), which promise potential applications in, e.g., deep ultraviolet light emitters (DUV LEDs), transparent membranes, protective coatings and dielectric layer^1^,^2^. h-BN consists of alternating *sp*^2^ hybridized B and N atoms within a single layer and week Vand de Waals interaction between stacking multilayers^3^. The advanced properties such as low dielectric constant, high temperature stability and high thermal conductivity has taken advantage over graphene^4^,^5^,^6^. h-BN also shows high oxidation resistant ability and can remain pristine at a considerably higher temperature over 800 °C^7^. Recently, it has been widely used as an important dielectric substrate to improve the electrical mobility of graphene based devices thanks to the high surface smoothness and the absence of dangling bonds^7^,^8^,^9^. In addition, the h-BN monolayer can be applied as an ideal substrate for the overgrowth of graphene directly and epitaxially, due to the small lattice mismatch of only 1.6%. Most recently, Kobayashi and his coworkers proposed a micromechanical separation technique for GaN epilayer of blue light LEDs by inserting h-BN as mechanical release layer^10^. All these applications of h-BN in electrical and/or optoelectronic devices strongly require a large size synthesis of h-BN film within a size limited reaction chamber, which is usually a horizontal tube.

Recently, a variety of methods have been developed to obtain atomically thin h-BN nanosheets on metal substrates. They include micromechanical cleavage^11^, pulsed CO_2_ laser deposition (PLD)^12^, and atmospheric pressure and low pressure chemical vapor deposition (CVD) methods. Among these methods, LPCVD is regarded the most stable technique for synthesizing high quality h-BN monolayer on the surface of (100) copper foil^13^, or even on various metal substrates^14^,^15^,^16^. Usually, the LPCVD furnace holds a horizontal quartz tube in limited diameter, which is the critical limitation restricting the scale-up of h-BN film synthesis. Enlargement of quartz tube diameter up to 8 inches has been reported for the growth of large area graphene films over 30 inches^17^. However, no matter how large the tube one tried to make, the tube size always remains an unsurpassable limit for material growth. How to overcome this limit and achieve extremely large size synthesis of h-BN films has been a very challenging issue.

Herein, we propose a novel technique with transforming the shape of Cu foil substrates to perform large-area roll synthesis of monolayer or even multilayer h-BN film by low pressure CVD method. By winding the Cu foil substrate from flat 2D film into quasi-3D mainspring shape, an extremely large size monolayer h-BN film of over 25-inch could be achieved with a reaction chamber in 1.2-inch radius. With the aid of PMMA, the h-BN monolayer was uniformly and smoothly transferred onto 2″ GaN or 4″ Si wafer surface. The robust antioxidant ability and monolayer feature of the h-BN layer was systematically characterized. Thanks to the weak out-of-plane interaction, the h-BN film was introduced as a self-release buffer layer on the GaN template. By hydride vapor phase epitaxy (HVPE) method with ammonia pre-treatment, the overgrowth of a 2″ thick GaN wafer over 200 μm has been successfully achieved above the h-BN buffer free of misfit strain.

## Results and Discussion

**Figure 1:**
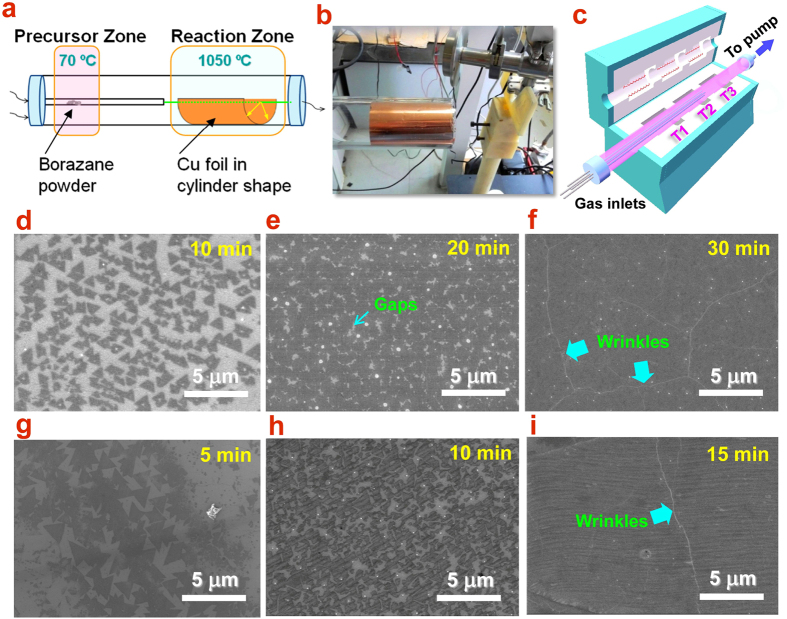
(**a,c**) Schematics of the LPCVD system for the growth of large-size monolayer h-BN. Borazane is put in a quartz boat of the precursor zone and the Cu foil is curved in cylindrical shape in the reaction zone. (**b**) Photograph of cylindrical Cu foil inserted into the reaction chamber, a 2″-diameter quartz tube. SEM images of as-grown h-BN flakes on Cu foil with a growth time of (**d**) 10 min, (**e**) 20 min, and (**f**) 30 min at 1000 °C; and (**g**) 5 min, (**h**) 10 min, and (**i**) 15 min at 1050 °C.

To understand the growth of h-BN in a LPCVD system, we conducted a systematic study on various critical parameters, in an attempt to achieve well-controlled h-BN flakes and coalesced films. We will focus on the effects of precursor temperature (T1), reaction temperature (T3), growth duration, and Cu lattice orientation. The above parameters are of potential importance to synthesize high quality uniform h-BN films. The domain sizes and film coverage can be controlled by varying the growth time. Figure 1d–f shows the increase of domain size and film coverage of h-BN at 1000 °C for three different growth times 10, 20 and 30 min, respectively. Triangular-shaped h-BN flakes can be clearly identified as darker region in Fig. 1d, and this well-defined shape is an indication of single-crystal h-BN domains as observed in previous work^8^. When increasing the growth time from 10 to 20 min, the monolayer h-BN domains ripen, coalesces, and form continuous films covering the Cu surface. As shown in Fig. 1e, it is worth noting that adjacent monolayer domains still have some gaps. After a longer growth time (30 min), h-BN domains go into a continuous film fully covering the Cu substrates, as shown in Fig. 1f. This can be identified by the presence of wrinkles due to the difference in thermal expansion coefficients temperatures of h-BN and Cu foil during cooling. SEM images in Fig. 1g–i show the evolution of the h-BN domains and coverage at higher T3 (1050 °C). It can be seen clearly that the slight temperature increase results in a significant improvement of the average and domain size, by comparing Fig. 1d,g. In light of this, we can conclude that higher growth temperature and shorter the deposition time should useful for obtaining the lower coverage and large domain size, which is essential for high quality h-BN films.

Although the growth of h-BN film can be achieved on Cu foil by CVD method, the film size is still limited by the diameter of quartz tube or the size of chamber. In order to extend the growth to larger area, we propose a method of deforming the Cu foil into quasi-3D shapes to overcome the chamber size limit. In practice, the Cu foil was wound into a cylindrical shape fitting the inner surface of a quartz tube, as shown in Fig. 1a. The inner small tube for carrying precursor was set aligned with the central axis of the cylinder. In this manner, the precursor could be carried to the surface of the cylindrical Cu foil uniformly in a radially symmetrical shape. The circumference of a cylinder can be obtained by 2*πr*, where *r* the radius of the quartz tube (>1.2 inch in our case). Thus, the inner surface actually could provide a large area up to 7 inches (7 × 5 in^2^).

**Figure 2:**
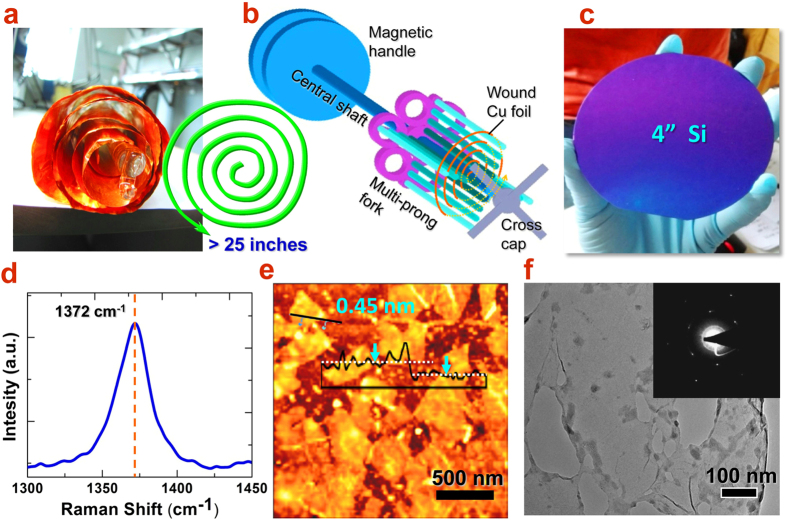
(**a**) Photograph of large-size Cu foil wound in mainspring shape and supported by a quartz fork. The schematic wreath shows the way of winding and the length of Cu foil is over 25 inches. (**b**) Schematic of multi-prong quartz fork equipped with a magnetic manipulator. The cross cap is designed for fixing the wound Cu foil during the manipulation inside quartz tube. (**c**) Smoothly transferred h-BN monolayer film on 4″ Si wafer. (**d**) Raman spectra of a h-BN thin film. (**e**) AFM image of transferred triangular h-BN domains with its corresponding height profile. (**f**) TEM image of h-BN monolayer. The inset shows an hexagonal SAED pattern of the h-BN monolayer.

Extending from the above curling technique, we further propose the mainspring-shaped winding of Cu foil to achieve unlimited size growth of h-BN film in a limited size quartz tube, as illustrated in Fig. 2a. Although the quartz diameter is fixed, the number of winding layers of Cu foil in principle could be infinite and only depends on how much spacing to leave between neighboring layers. Therefore, the reactor size limit could be completely overcome by extending the area to a continuous 2D curl of plane inward. Suppose that a 5-layer wound Cu foil roll is made for a 1.2″ tube, the unwrapped area of the available growth surface for h-BN growth could reach over 25 inches. In order to avoid the touching of neighboring foil layers and to keep uniform intervals, a multi-prong quartz fork tool was designed to support the wound spring-shape Cu foil, as shown in the schematic of Fig. 2b. This tool consists of three important functional sections: magnetic handle, multi-prong fork and cross cap. The magnetic handle could be combined with a magnetic ring, with which the manipulation of the fork (push, drag and rotate) from outside the tube becomes possible by controlling with another magnet block (See details in Supporting information). The multi-prong fork includes several sets of prongs in a cross shape. Two prong rows are perpendicular to each other so that prongs could provide support of wound foil and maintain its circularity. The Cu foil could be easily wound into these prongs layer by layer and meanwhile, the space between layers is left wide enough for the carrier gases smoothly flowing through. Finally, a cross cap is used to lock the tip of the fork, which could fix the foil roll and allow the movement of the foil together with entire fork when the magnetic manipulation is applied. With the aid of this multi-prong fork, a large-roll growth of h-BN film was grown and the properties were investigated.

The chemical composition, atomic bonding and structural properties of the as-grown h-BN film were investigated by using Raman spectroscopy, AFM and TEM. After transfer on to SiO_2_/Si substrate, a highly symmetric Raman peak can be recorded located at about 1372 cm^−1^, as shown in Fig. 2d. This characteristic peak confirms the presence of the h-BN monolayer, corresponding to the E_2g_ vibration mode of hexagonal B–N bonds. As reported previously, the presence of decomposition byproducts of borazane in samples, such as c-BN, B_x_C_y_N_z_, BN soot or multilayer h-BN film, usually lead to asymmetric Raman peaks that can be further deconvoluted into a 1370 cm^−1^ peak and some other small peaks at lower frequencies^18^,^19^,^20^. Therefore, the symmetric Raman G band at 1372 cm^−1^ demonstrates the high quality of the hexagonal h-BN monolayer.

On the other hand, the AFM image of the triangular h-BN flakes transferred onto the SiO_2_/Si substrate was taken and shown in Fig. 2e. The thicknesses of the flakes can be determined to be 0.45 nm, corresponding to a single atomic layer. It was also observed that the coalesced neighboring h-BN domains were seamlessly stitched in a uniform height profile and forms the continuously complete monolayer film. Above monolayer film, the growth of the second layer starts and then the bi- or multi-layers of h-BN film could grow one after another. The crystalline structure of h-BN domains often shows up in a triangular shape mainly due to the asymmetric formation energy of the B- or N-terminated edges. In principle, the N-terminated edges are energetically more favorable so that three equivalent N-terminated edges will enclose a stable triangular-shape domain. The structural information of the h-BN thin film was characterized by HRTEM and SAED, as shown in Fig. 2f. The LPCVD grown h-BN films were first transferred onto copper TEM grids coated with carbon support film, as shown by the low magnifcation TEM image. From the contrast to empty holes, the suspended h-BN film could be clearly distinguished. The electron diffraction patterns were taken from different regions of the film (inset of Fig. 2f). The SAED clearly shows a set of hexagonal diffraction spots matched well with the (10-10) index of monolayer h-BN, indicating the highly crystalline structure.

**Figure 3:**
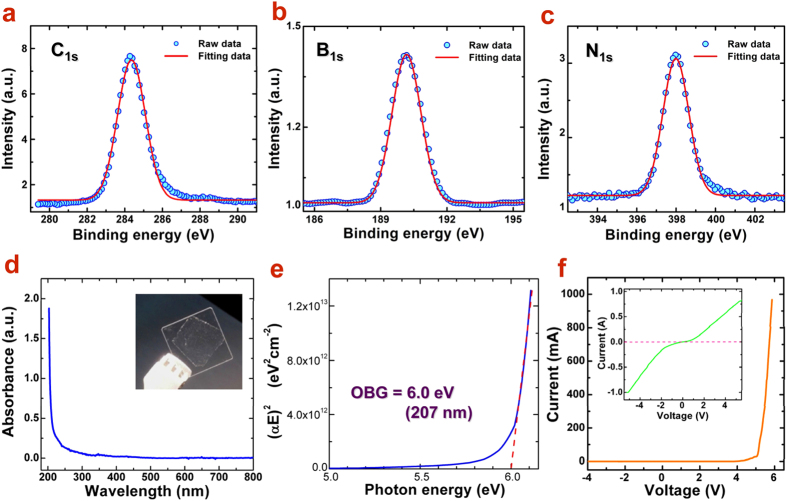
(**a–c**) XPS spectra of h-BN monolayer transferred on SiO_2_ substrate, showing C_1s_, B_1s_, and N_1s_ core levels, respectively. The peaks of (**a–c**) were fitted with Gaussian curves (red peaks). (**d**) UV−Visible absorption spectrum of h-BN film on quartz substrate (inset) measured at room temperature, and (**e**) Tauc’s plot of (αE)^2^ versus energy (E) for determing the optical bandgap (OBG) of h-BN film, which is about 6.0 eV. (**f**) I-V curve of the monolayer h-BN device on n-Si substrate, indicating the highly insulating nature below 5.0 V. The inset shows the I-V curve with two-probe measurement on bare n-Si surface.

X-ray photoelectron spectroscopy (XPS) was also applied to determine the elemental stoichiometry of the synthesized h-BN film. Figure 3(a–c) shows the XPS spectra of the h-BN thin film on a SiO_2_/Si substrate, which is consistent with that from the as-grown h-BN film on Cu foil (See supporting information). The peak energies of N_1s_ and B_1s_ were corrected with respect to the binding energy of the C_1s_ peak (C–C bond) at 284.5 eV. Thus, the observed binding energies of N_1s_ and B_1s_ peaks are 398.0 and 190.2 eV, respectively, in good agreement with the literature values^21^,^22^. These binding energies are directly related with the hexagonal B–N bonding, implying the hexagonal phase of the BN film. In principle, the characteristic peak of B–O should appear at higher energy around 192 eV. In our LPCVD system, it provided a vacuum <10^−4^ Torr and a pre-annealing under Ar/H_2_ flow was carried out to remove the oxides on Cu foil surface. Hence, it is believed oxidation in the h-BN film could be excluded. The B/N stoichoimetric ratio from our XPS survey was calculated to be 1.06, close to perfect 1:1. The additional carbon peak could result from the exposure of the h-BN film to air during transfer processes. Therefore, the purity of h-BN confirms the correct hexagonal coordination and the high crystalline quality. The antioxidant and protective abilities of h-BN film was also examined by heating the BN-covered Cu foil in air at 200 °C (Fig. S10). It was found that the Cu foil surface could be well protected from oxidation, which revealed the potential application of h-BN as protective coating layer for metals^23^.

In order to determine the optical bandgap (OBG) *E*_*g*_, We have tried to measure the emission peak of the as-grown h-BN monlayer by using photoluminescence and cathodoluminescence, respectively. However, due to the ultrathin of the monolayer and the extremely week absorption and emission, no obvious signal was detected from the h-BN monolayer. Hence, the UV-vis spectrophotometer was employed to record the transmittance and absorption spectrum instead. First, the h-BN film was transferred onto a quartz substrate, which is highly transparent from 199 nm and up. By using a blank quartz substrate for baseline subtraction, a strong absorption peak appears at 207 nm, as shown in Fig. 3d. For direct bandgap semiconductor, the absorption coefficient can be defined as α = *A*(*E* − *E*_*g*_)^1/2^/*E*, where *A* is a constant and *E* stands for the photon energy. In Fig. 3e, the (α*E*)^2^ as a function of *E* is plot and the extrapolation of a straight line of the energy dispersion curve to intersect the *E*-axis gives the value of *E*_*g*_. With this method, the OBG of monolayer h-BN film is determined to be 6.0 eV, which agrees well with previous measured values ranging from 5.84 to 6.07 eV^19^,^24^. The measured wide OBG suggests the high transparency and good electrical insulation of h-BN film and hopefully, broad applications in short-wavelength optoelectronic devices.

The wide bandgap usually will also correspond to an insulating (dielectric) property of the film. In order to perform electrical measurements, the h-BN layer was transferred on to an n-type Si substrate and the electrical characteristics were further measured by using four-point probe technique to confirm its insulating feature. Since the n-type Si substrate possesses low resistivity (<1 Ω cm), the dielectric behavior of h-BN could be easily distinguished. Figure 3f shows the obtained I-V curve for h-BN film in which zero current holds over a wide voltage range up to 5 V. This turn-on voltage is related with the bandgap (~6 eV). For comparison, the I-V curve of the beneath *n*-Si surface shows a highly conducting behavior (inset). This strongly confirms the excellent insulating property as well as the high crystalline quality of the as-grown large area h-BN film, which is also indicative of potential applications as dielectric layer for two dimensional devices in conjunction with graphene^25^,^26^,^27^.

**Figure 4:**
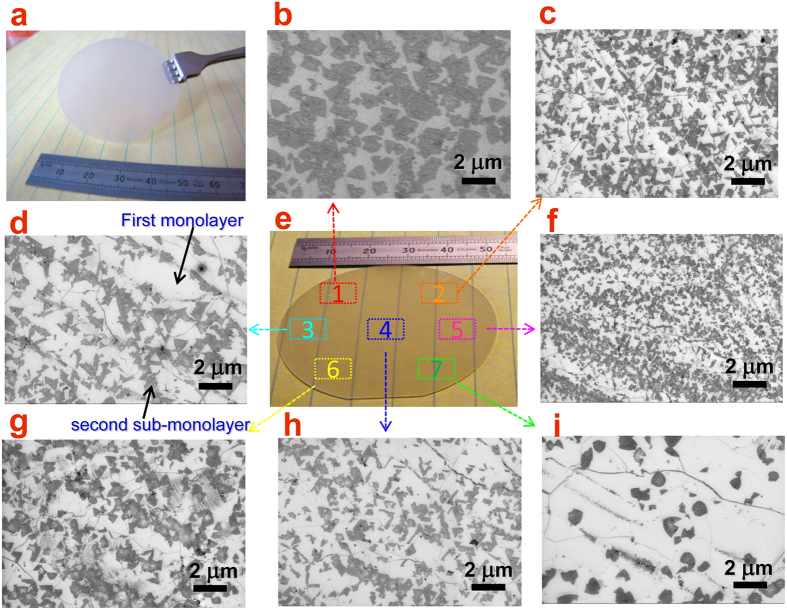
(**a**) Photograph of the transferred and well bonded h-BN film on a 2″ GaN/sapphire full wafer. (**b–i**) SEM images of h-BN covered GaN surface in different regions, as indicated in (**e**). These show the completeness and uniformity of the h-BN film after transfer.

Such a large area h-BN is now very suitable for wafer-scale applications on semiconductor wafers. However, the stable transfer method plays the critical role among these processing techniques. To improve the transfer process, a set of specific transfer tools such as flat spades with handles in different angles were designed to carry and move h-BN films (See also Supporting information). The holes on the spade could filter the solution and minimize the liquid resistance. Thus, the h-BN film carried by this spade could be transferred more smoothly. With the aid of PMMA, the large-size h-BN layer could be stably transferred onto the surface of a 2″ GaN/sapphire wafer, as shown in Fig. 4a, or even a 4″ Si wafer (inset of Fig. 2c).

The purpose of the introduction of h-BN buffer layer for the GaN overgrowth is to enhance the mechanical self-release of GaN thick layer or to induce the release of residual misfit strain on epilayers. Concerning about the weak Van der Waals interaction between h-BN stacking layers, a 1.x monolayer h-BN film (complete first layer and sub-mono second layer) was grown based on the monolayer h-BN film by increasing the growth time to initiate the second sub-monolayer formation. As a result, triangular flacks in the second h-BN layer could be obtained and the loose interaction between beneath monolayer and upper sub-monolayer is what we need for strain release in GaN thick layer. Hence, this 1.x monolayer h-BN film was chosen as the buffer on GaN wafer for proceeding GaN epitaxy. The h-BN film is tightly and flatly bonded with the GaN (0001) surface, showing an optically smoothness. SEM images were taken on different positions over the entire 2″ wafer surface, as shown in Fig. 4b–i. One can see that the triangular-shape h-BN domains on the second layer appear darker due to its insulating character and thus can be clearly identified. The white background with well spreading wrinkles indicates the full coverage with continuous first layer. The uniform distribution of triangular h-BN domains suggests the complete coverage and uniformity of the h-BN film on GaN wafer. This provides the important basis of high-quality h-BN buffer layer for the following overgrowth of GaN epilayer.

**Figure 5:**
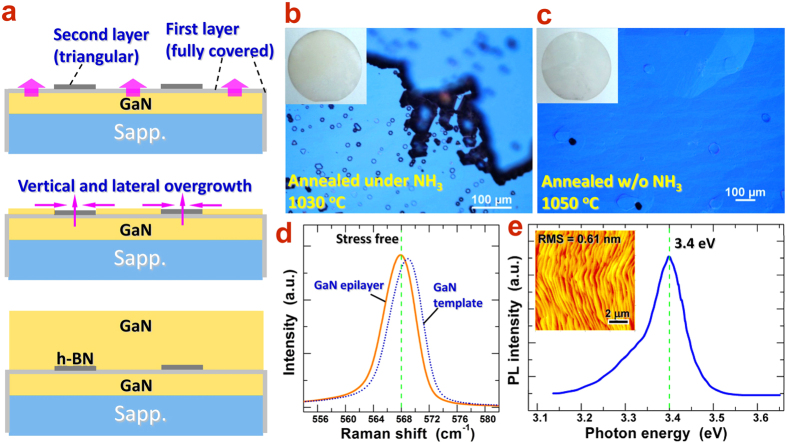
(**a**) Schematic diagram illustrating the overgrowth process of GaN thick epilayer on the h-BN buffered GaN/sapphire template. Directly vertical overgrowth and lateral growth finally lead to the uniform GaN thick epilayer. Morphology of the overgrown GaN epilayer by HVPE with pre-annealing under NH_3_ (**b**) and in the absence of NH_3_ flow (**c**). The insets show the photographs of as-grown GaN thick wafers. (**d**) Raman spectra of GaN epilayer grown on BN/GaN/sapphire substrate (solid) and GaN/sapphire (dotted), showing the good release of residual strain by the aid of h-BN buffer. (**e**) PL Spectrum of as-grown GaN thick epilayer. The inset shows the AFM image of smooth GaN surface (RMS = 0.61 nm).

The overgrowth of GaN thick wafer was carried out by using HVPE method. Figure 5a shows the schematic diagrams illustrating the vertical and lateral overgrowth processes of GaN epilayer above h-BN buffer. Since the hBN monolayer is very thin, the GaN epitaxy could be favored upon the first layer vertically and then, the lateral overgrowth together with slow vertical growth takes place on the second-layer h-BN flakes. After complete coalescence and continuous growth, a uniform GaN thick epilayer could be achieved, as shown in the lower part of Fig. 5a. As we know, wrinkles or ripples of h-BN may be present after the transfer onto GaN template. Fortunately, the HVPE growth of GaN epilayer is at high temperature (1050 °C). The GaN/Sapphire will undergo a considerable thermal expansion, which will make the wrinkles flat and minimize the height of remaining ripples. Thus, the overgrowth of GaN epilayer could easily extend onto the ripple ridges and coalesce gradually (see also supporting information). In practice, the main factors affecting the overgrowth include the pre-annealing ambient and the growth temperature. For comparison, a series of GaN epitaxial samples were grown on BN/GaN/sapphire templates at 1030~1050 °C pre-annealed under NH_3_ and in the absence of NH_3_, as shown in Fig. 5b,c. We find that the annealing atmosphere is critical for the nucleation of GaN over h-BN buffer. From Fig. 5b, one can see that the presence of NH_3_ during the pre-annealing and lower growth temperature at 1030 °C easily lead to the yellowish color and roughness of the epilayer surface, indicating the significant impurity incorporation and defect formation. This may be attributed to the over-nitridation of the h-BN/GaN interface, which could change the Ga-terminated face to partially N-terminated one. Thus, the impurities incorporation could be energetically favored and the molecule migration on the surface is slightly depressed. As a result, the crystal quality of the epilayer decreases.

In contrast, the epitaxial layer after pre-annealing under vacuum and under increasing growth temperature (1050 °C) is apparently improved even by naked eyes, as shown in the inset of Fig. 5c. The optical images show the high transparency and smoothness of the epilayer surface. It was found that the annealing in vacuum could effectively remove the organic residues involved from the h-BN transfer process and preserve the Ga-terminated interface between the h-BN buffer and the underlying GaN surface. The Ga-face interface could effectively enhance the nucleation of new epilayer above the h-BN buffer and maintain the high crytal quality as well. The higher growth temperature in principle will provide high migration energy of molecules (Ga-N) on the surface and hence, enhance the lateral overgrowth rate above the second h-BN layer. Finally, a successful overgrowth of smooth and thick GaN epilayer has been achieved over 200 μm above h-BN buffer.

In order to characterize the residual strain state, Raman measurements were carried out for the GaN epilayer surface as well as the GaN template. Figure 5d shows the characteristic Raman spectrum of GaN in the E_2_ (high) mode, which is at about 568.0 cm^−1^. This value is well consistent with that of free-standing bulk GaN^28^, confirming the effective stress release after the introduction of h-BN buffer layer. In contrast, the Raman peak of GaN template locates at 569 cm^−1^, indicating the subjection to a compressive stress. As we know, the out-of-plane Van de Waals forces between h-BN stacking layers leads to the week interlayer interaction. When the h-BN buffer is inserted into the interface between the underlying and overgrowth GaN epilayers, the existing residual misfit strain extending from sapphire substrate would be suddenly blocked and released. Thus, the upper overgrown GaN thick epilayer can be subjected to a state free of compressive stress. To further investigate the optical properties, PL measurement was carried out to examine the energy shift of photons. As shown in Fig. 5e, a sharp PL peak can be seen centered at 3.4 eV, which is exactly the optical bandgap of bulk GaN under zero-stress. Another small shoulder at around 3.32 eV should come from the GaN template, red-shhifted due to the compressive strain. Finally, AFM scanning of the topmost surface shows a roughness of rms = 0.61 nm (inset of Fig. 5e), indicating a considerably smooth surface by HVPE growth. The XRD rocking curve scan gives an estimation of the dislocation density in the order of 10^6^ cm^−2^.

## Conclusions

In conclusion, we have achieved the large-roll synthesis of 25-inch monolayer h-BN film with a method of winding Cu foil substrates into quasi-3D shapes by LPCVD growth. By winding the Cu foil from flat 2D surface into cylindrical and mainspring shapes supported with a multi-prong quartz fork, the reactor size limit could be overcome by extending the substrate area to a continuously curled 2D plane inward, which actually could be infinite. Hence, unlimited size h-BN film can be grown in a 1.2-inch quartz tube. The OBG of h-BN monolayer was determined to be 6.0 eV. The complete h-BN sheet showed a robust antioxidant ability after continuous heating in air for 2 hours. With the aid of PMMA protection, the h-BN film was uniformly transferred onto 2″ GaN or 4″ Si wafer as a self-release buffer. After pre-annealing under vacuum, the overgrowth of a 2″ thick GaN wafer over 200 μm has been successfully achieved above the bilayer h-BN buffer by HVPE method. Due to the week Van de Waals interaction of h-BN layers, complete release of misfit strain leads to a stress-free GaN thick epilayer for future applications as free-standing wafer.

## Methods

### LPCVD set-up and synthesis of h-BN film

The growth of h-BN films was carried out by setting up a LPCVD system with 3 heating zones and 4 independent gas lines. Figure 1a–c show the schematic diagram of LPCVD used in our work. Solid borazane was used as precursor of source of B and N and put in precursor zone. Cu foils in 25 μm thickness was employed as the substrate and catalyst for h-BN growth and put in reaction zone. The zone in between was used to heat the borazane vapor on the way to reaction zone, preventing deposition of particles on Cu substrate. Firstly, the Cu foil was placed in the reaction zone and heated up to 800 °C at low pressure (<10^−4^ Torr). Annealing of Cu foil was carried out for 30 min in an argon/hydrogen flow for removing the surface oxides and enlarging the grain size. Secondly, the chamber was continuously heated up to ~1050 °C for the growth of h-BN. Meanwhile, the precursor was heated at about 80 °C to generate borozane vapor, which is carried by Ar/H_2_ mixed gases into the reaction zone. The typical CVD growth time was 5~60 min. After the growth, the chamber was cooled down to room temperature and the sample was kept protected by Ar gas flow. Within a range of temperatures (for example, 80~100 °C in our case), ammonia borane molecules dissociate into three products: hydrogen, polyiminoborane (BH_2_NH_2_; solid), and borazine ((HBNH)_3_; gas). Borazane molecules provide the main building blocks for the growth of h-BN on the Cu surface under high temperature.

### Transfer method of h-BN film

As-grown h-BN film could be transferred on various substrates using the PMMA assisted wet chemical etching process (See also supporting informaion). (i) A thin layer (~1 μm) of 5% PMMA diluted in anisole was spin-coated onto the h-BN/Cu surface. Deposition of uniform thin PMMA layer can well support and protect the h-BN layer from breaking during the following etching and washing processes. (ii) The PMMA/h-BN/Cu sample was baked at about 170 °C for 10 min and cooled down to room temperature. (iii) The Cu substrate etching was done with aqueous (NH4)_2_S_2_O_8_ solution gradually and then, only the h-BN/PMMA film was left floating on the solution surface. (iv) DI water washing was employed to remove contaminations from the (NH_4_)_2_S_2_O_8_ solution. (v) The h-BN could be transferred onto any target substrate, e.g., Si, SiO_2_/Si, quartz glass, or transmission electron microscopy (TEM) grids etc. (vi) Acetone was employed to remove PMMA. (vii) Annealing at around 300~500 °C under high oxygen flow for 10 min was carried out to further remove the PMMA residues. Finally, the complete and perfect transfer of the monolayer h-BN film could be accomplished.

### HVPE overgrowth of GaN wafer

For the overgrowth of GaN thick epilayer, a 3-μm-thick GaN layer was first grown on sapphire substrate by MOCVD as a template. h-BN film was uniformly transferred onto 2″ GaN/sapphire template with the method described above. Then, thick GaN layer re-growth was carried out by HVPE system. Prior to growth, the h-BN coated GaN template was cleaned by acetone and methyl alcohol. After that they were overflowed with DI water for 5 min. The growth system was equipped with a quartz horizontal reactor under atmospheric pressure. HCl and NH_3_ were used as precursors. In HVPE reactor, HCl gas reacted with liquid Ga to form GaCl gas, which was transported to the growth zone of the reactor and reacted with NH_3_ to form GaN deposition on h-BN/GaN template. The template was loaded into tube furnace and pre-annealed with or w/o ammonia flow for 3 min. Then the GaN overgrowth was carried out at a temperature of 1050 °C. It is believed that most PMMA residues are removed. The remaining tiny residues were carbonized and the influence on the GaN epilayer could be an incorporation of C dopants in the GaN epilayer close to the hBN/GaN interface.

### Characterization

The Normarski optical microscopy (Olympus BX51M) was employed to observe the morphology of Cu surface before and after h-BN growth, and the surface of as-grown GaN thick wafer. The structure and coverage of h-BN film were characterized by scanning electron microscope (SEM, Hitachi S-4800 scanning electron microscope operated at 20 kV). The Raman spectra and spectral mapping were recorded for investigating the characteristic resonance of BN bonds or determining the stress of GaN thick layers, using a Renishaw InVia Raman Microprobe equipped with a 532-nm laser. The laser spot is about 1~2 μm in diameter on the sample. The spectrum was taken by averaging over 5 circles. h-BN films were carefully transferred onto TEM grids coated with carbon support film. The TEM investigations were carried out using a Philips Tecnai F30 field-emission electron microscope with an accelerating voltage of 200 kV. The selected area electron diffraction (SAED) pattern was recorded to confirm the monolayer and crystalline structure of h-BN film. Atomic force microscopy (AFM) with a Veeco system was employed to measure the morphology and thickness of h-BN flakes or complete film. X-ray photoelecton spectroscopy (XPS) (PHI Quantera XPS) was performed on h-BN/Si samples using monochromatic copper Kα X-rays, and the MultiPak software was used for data analyses and fitting. High-resolution X-ray diffraction (XRD) measurements were carried out to qualify the crystal quality of GaN wafer, using a Bruker D8 Discovery system. The room temperature Photoluminescence (PL) was carried out to determine band-edge emission and the inference by residual strains.

## Additional Information

**How to cite this article**: Wu, C. *et al*. Large-roll growth of 25-inch hexagonal BN monolayer film for self-release buffer layer of free-standing GaN wafer. *Sci. Rep.* **6**, 34766; doi: 10.1038/srep34766 (2016).

## Supplementary Material

Supplementary Information
