# Supplementary material for: Large-roll growth of 25-inch hexagonal BN monolayer film for self-release buffer layer of free-standing GaN wafer
Source: Sci Rep. 2016 Oct 19;6:34766. doi: 10.1038/srep34766 (PMC5069463; doi:10.1038/srep34766)
Supplement: Supplementary Information [file srep34766-s1.pdf]

Supporting Information for :

Large-roll growth of 25-inch hexagonal BN monolayer film for self-release buffer layer of free-standing GaN wafer

Chenping Wu [1], Abdul Majid Soomro [1], Feipeng Sun [1], Huachun Wang [1], Youyang Huang [1], Jiejun Wu [a),2], Chuan Liu [3], Xiaodong Yang [3], Na Gao [1], Xiaohong Chen [1], Junyong Kang [1], and Duanjun Cai [a),1,4]

[1] *Fujian Key Laboratory of Semiconductor Materials and Applications, CI center for OSED, College of Physical Science and Technology, Xiamen University, Xiamen 361005, China.*

[2] *Research Center for Wide-gap Semiconductors, State Key Laboratory for Artificial Microstructures and Mesoscopic Physics, School of Physics, Peking University, Beijing 100871, China.*

[3] *State Key Laboratory of Physical Chemistry of Solid Surfaces, College of Chemistry and Chemical Engineering, Xiamen University, Xiamen 361005,China.*

[4] *Department of Chemistry, Duke University, Durham, NC 27708-0354, U.S.A.*

a) E-mail: dcai@xmu.edu.cn, wujiejun@pku.edu.cn

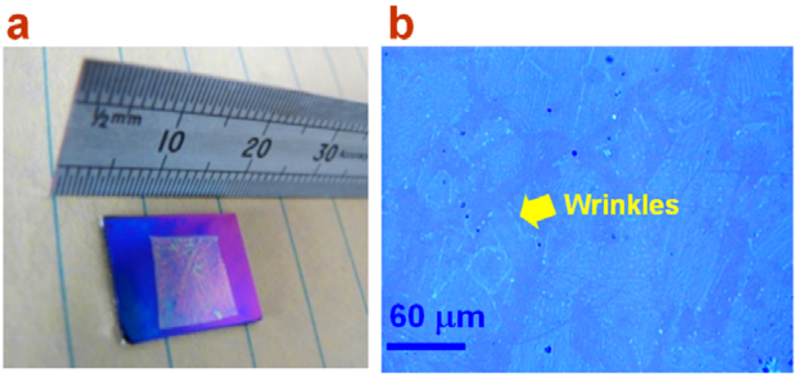

Fig. S1. Photograph (a) and optical micrograph (b) of transferred h-BN film on Si substrate. Clear wrinkles indicate the fully coverage of h-BN film on the surface.

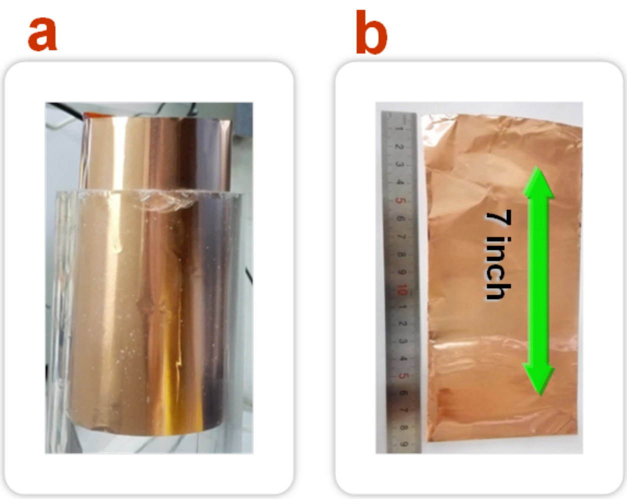

Fig. S2. Photographs of cylindrical-shape Cu foils. (a) Cylindrical Cu coil inserted in quartz tube, and (b) unwrapped Cu foil after h-BN growth, showing a large size of over 7 inches.

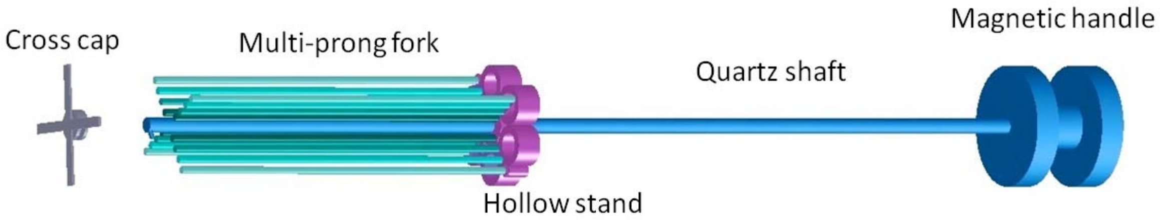

Fig. S3. Schematic sideview of multi-prong quartz fork equipped with a magnetic handle and cross cap. The hollow stand is designed for supporting the multi-prong fork and meanwhile, maintaining a fluent gas flow through the winded Cu foil surfaces.

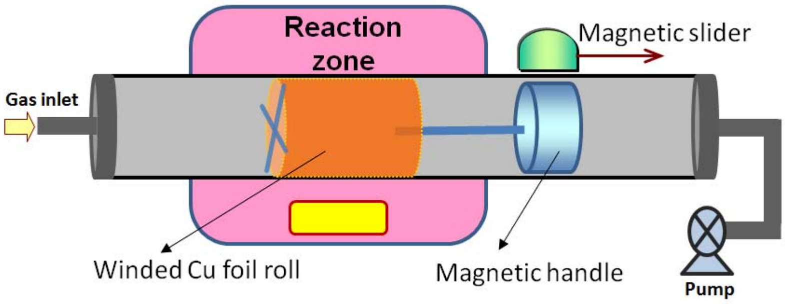

Fig. S4. Schematic of LPCVD system with magnetic manipulation of quartz fork. With a magnetic slider, operator could manipulate the fork (push, drag, rotate) in the vacuum chamber from outside conveniently.

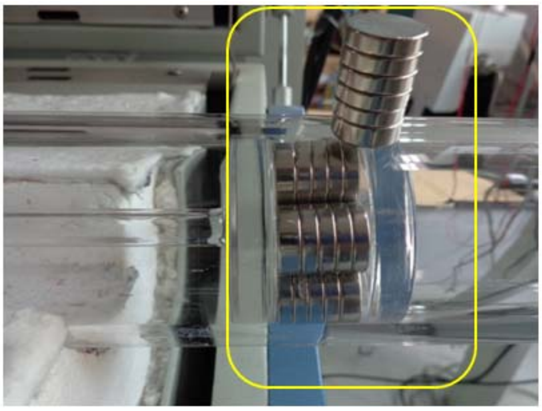

Fig. S5. Photograph of the magnetic handle and manipulator, which can be controlled from outside the quartz tube.

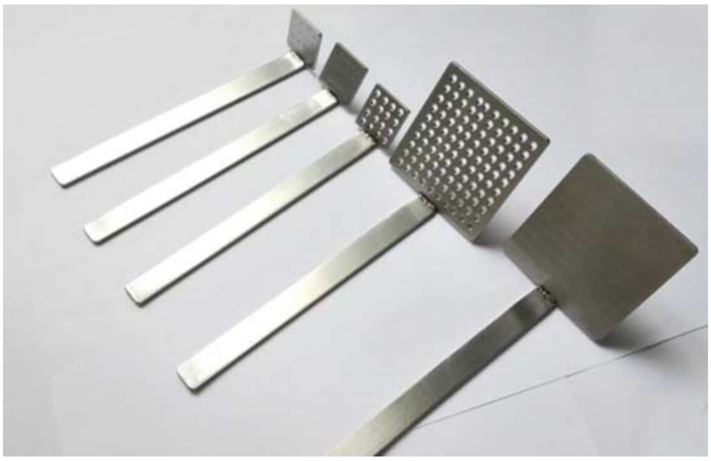

Fig. S6. Photograph of a set of home-made spade tools for h-BN film transfer.

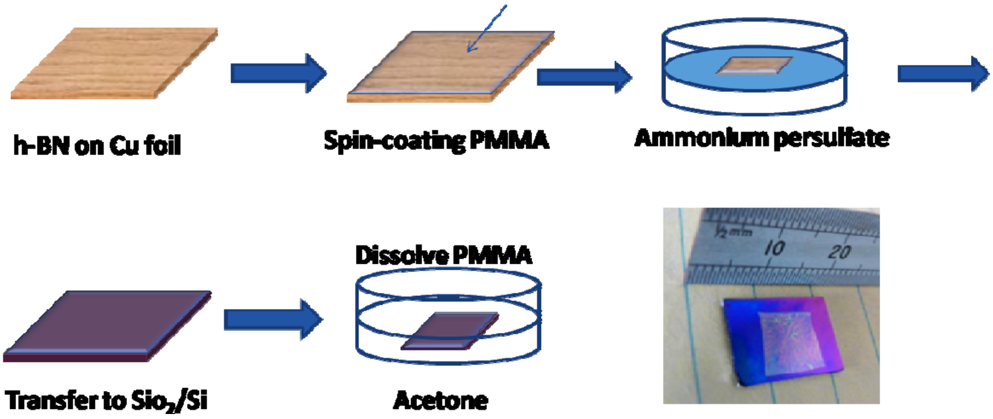

Fig. S7. Schematics of the PMMA-assisted transfer method for h-BN film.

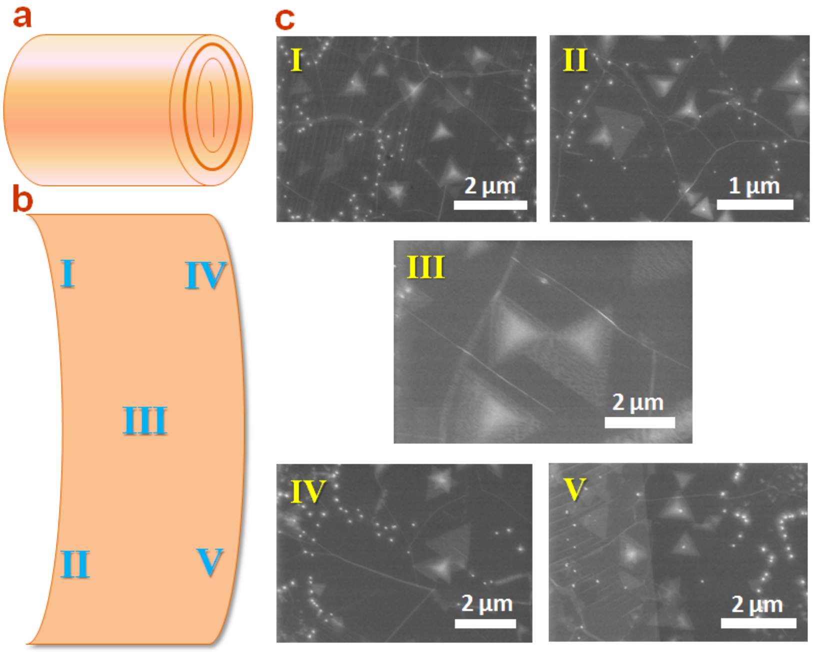

Fig. S8. Schematics of (a) winded string-shape Cu foil curl and (b) unwrapped Cu foil after h-BN synthesis. Representative positions on the Cu foil are marked. (c) SEM images of as-grown 1.x monolayer h-BN film at different positions as marked in (b). This confirms the uniformity of h-BN growth on the winded Cu foil surface over a large area.

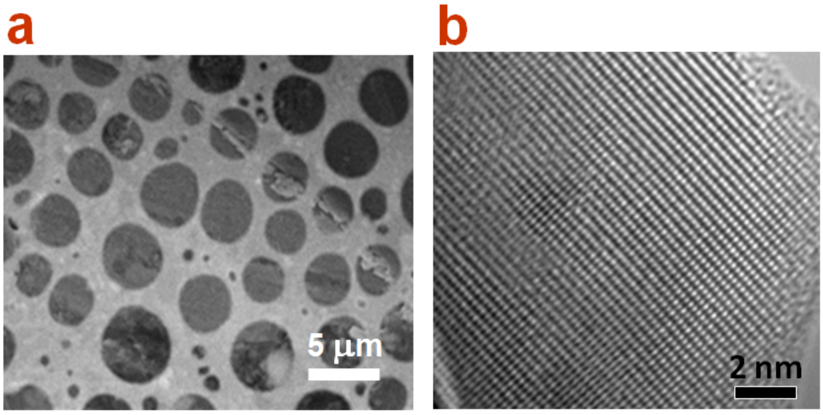

Fig. S9. Low resolution (a) and high resolution (b) TEM images of transferred h-BN layer. Hexagonal single crystalline structure could be clearly seen over a large area.

The antioxidant ability was examined by heating the sample in air at 200 °C for 20 min. We put two samples with different growth time, 10 min and 30 min, respectively, on the hot plate. After heating for 20 min, one can find that the partially covered sample shows serious oxidization by the air, exhibiting a color change from yellow to light-red and purple. This indicates the formation of CuO. In contrast, the fully covered sample with 30 min growth of h-BN preserves its original color of Cu foil beneath h-BN. This reflects two important feature of h-BN film, one is the transparency with monolayer thickness (wide band gap) and another is its

strong antioxidant ability. Further application of h-BN as protecting layer on other material against oxidation could be carried out.

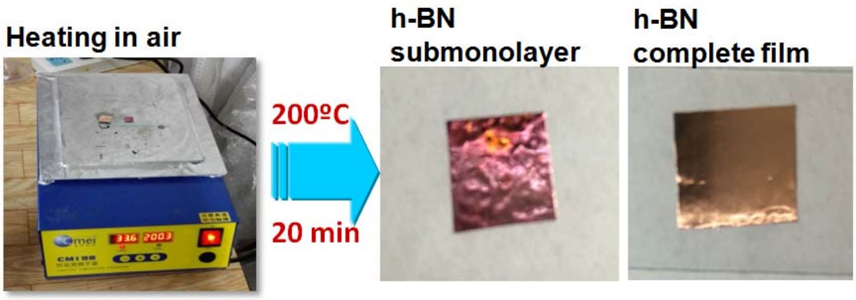

Fig. S10. Anti-oxidant ability test of h-BN film by heating in air. The complete h-BN film shows robust anti-oxidant capability.

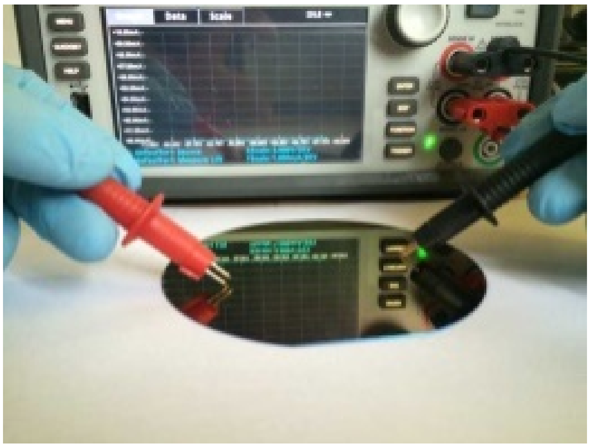

Fig. S11. Photograph of electrical measurements of h-BN/SiO_2 devices on 4” n-type Si wafer.

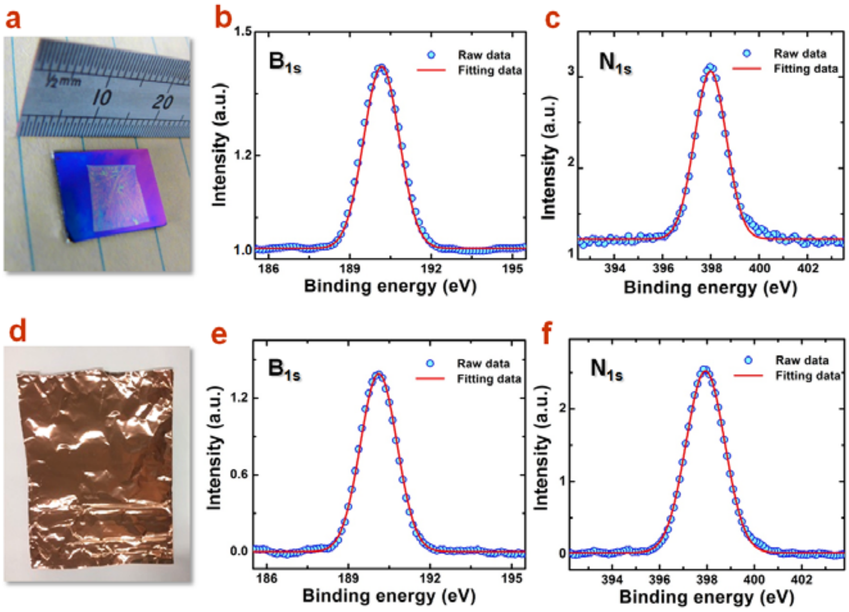

Fig. S12. XPS spectra of h-BN film on SiO2 substrate (a-c) and on Cu foil (d-f), showing B_{1s} and N_{1s} core levels, respectively.

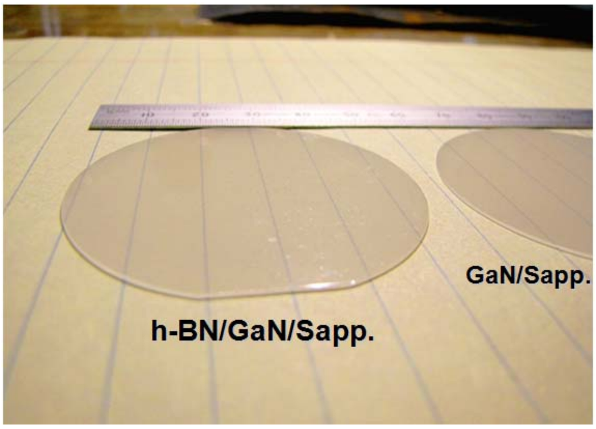

Fig. S13. Photograph of surfaces of 2” h-BN/GaN/Sapphire and bare GaN/Sapphire wafer.

As we know, after the oxidation, the PMMA residue could be largely removed and the surface becomes rather smooth, as we can see from the AFM image. Even though, some residues remain on the surface. Prior to the GaN overgrowth, another annealing process was carried out at much high temperature (> 1000 ^{o}C) in the reactor chamber for 3 min. Then the HVPE GaN overgrowth was carried out at a temperature of 1050 °C. Hence, it is believed that most PMMA residues are removed. The remaining tiny residues were carbonized and the influence on the GaN epilayer could be an incorporation of C dopants in the GaN epilayer close to the hBN/GaN interface.

A set of tools was designed for stably carrying, salvaging and transferring of h-BN film from various solutions, as shown in **Fig. S6**. These tools have a spade-like shape with holes on the plate, which could firmly support a substrate or h-BN film. Meanwhile, liquid could fluently flow away through these holes. In the critical step of covering h-BN/PMMA film onto target wafer surface, an extremely slow and steady salvage process was carried out via the control with a vertical slide rail. Meanwhile, the surface of the target wafer (GaN/sapphire) should be kept tilt by a small angle (~5°). In such manner, DI water will be slowly drained away from the wafer surface and the hBN/PMMA film could be smoothly come in contact with the surface with very few bubbles or ripples, as shown in **Fig. S13**. On the other hand, the HVPE growth of GaN epilayer is at high temperature (1050 °C). At this temperature, the GaN/Sapphire will undergo a considerable thermal expansion, which will make the wrinkles flat and minimize the height of remaining ripples. Thus, the overgrowth of GaN epilayer could easily extend onto the ripple ridges and coalesce gradually, as shown in **Fig. S14**. Finally, the strain-free GaN epilayer could be achieved.

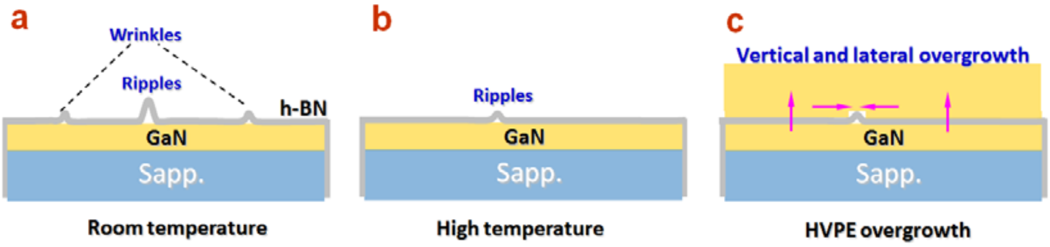

Fig. S14. Schematic of influences of h-BN winkles and ripples on GaN epilayer overgrowth.
